# Penetration of Piperacillin/Tazobactam Into Gynecologic Tissues Following a Single Loading Dose Prior to Hysterectomy

**DOI:** 10.1155/S1064744994000347

**Published:** 1994

**Authors:** William F. O'Brien, Shelley George

**Affiliations:** Division of Obstetrics University of South Florida College of Medicine Suite 506, 4 Columbia Drive Tampa FL 33606 USA

## Abstract

*Objective:* This study was undertaken to determine plasma and pelvic tissue concentrations of piperacillin/tazobactam following a single intravenous administration.

*Methods:* Plasma and tissue samples were obtained following administration of 3 g of piperacillin and 0.375 g of tazobactam. Concentrations of these agents were determined by chromatography.

*Results:* Plasma concentrations (mean ±SD) for piperacillin/tazobactam were 208 ± 87/27 ± 8 and 95 ± 53/14 ± 2, respectively, at 30 and 60 min after the start of the infusion. The 8:1 ratio between piperacillin and tazobactam in plasma remained constant for up to 96 min. Tissue concentrations yielded a broader range, but each agent achieved significant penetration in each of the tissues studied.

*Conclusions:* Piperacillin/tazobactam demonstrates favorable penetration in female genital tract tissues and Should be considered a potentially effective agent for female pelvic infections.


Infectious Diseases in Obstetrics and Gynecology 2:20–24 (1994)
© 1994 Wiley-Liss, Inc.

### Penetration of Piperacillin/Tazobactam Into Gynecologic Tissues Following a Single Loading Dose Prior to Hysterectomy

**William F. O'Brien and Shelley George**

*Division of Obstetrics, University of South Florida College of Medicine, Tampa, FL*

ABSTRACT

***Objective:*** **This study was undertaken to determine plasma and pelvic tissue concentrations of piperacillin/tazobactam following a single intravenous administration.**

***Methods:*** **Plasma and tissue samples were obtained following administration of 3 g of piperacillin and 0.375 g of tazobactam. Concentrations of these agents were determined by chromatography.**

***Results:*** **Plasma concentrations (mean ± SD) for piperacillin/tazobactam were 208 ± 87/27 ± 8 and 95 ± 53/14 ± 2, respectively, at 30 and 60 min after the start of the infusion. The 8:1 ratio between piperacillin and tazobactam in plasma remained constant for up to 96 min. Tissue concentrations yielded a broader range, but each agent achieved significant penetration in each of the tissues studied.**

***Conclusions:*** **Piperacillin/tazobactam demonstrates favorable penetration in female genital tract tissues and should be considered a potentially effective agent for female pelvic infections.**
© 1994 Wiley-Liss, Inc.

KEY WORDS
Pelvic infection, prophylaxis, antibiotic

The challenge of β-lactamase-mediated antibiotic resistance has led to the development of agents with increasing resistance to enzymatic inactivation. When combined with these β-lactamase inhibitors, antibiotics are effective against a broader spectrum of organisms.

Currently available inhibitors include clavulanic acid and sulbactam, which, in combination with amoxicillin, ticarcillin, or ampicillin, have been successful in treating infections caused by *Staphylococcus aureus, Bacteroids fragilis, Hemophilus influenzae, Escherichia coli,* and other gram-negative bacteria. Tazobactam, a new derivative of penicillinic acid sulfone, acts as an irreversible inhibitor of the β-lactamase from many bacteria.[1] Activity has been demonstrated against plasmid-mediated β-lactamases and chromosome-encoded cephalosporinases produced by Enterobacteriaceae. In an 8:1 dosage ratio, the combination of piperacillin and tazobactam has been markedly effective in the treatment of a variety of infections.[2]

An average of 2–4 aerobic and anaerobic organisms can be isolated from women with postpartum infection and endometritis.[3] Treatment is therefore based on the polymicrobial nature of the disease. In view of the varying clinical presentation and uncertain microbial nature of female genital tract disease, no agent with sufficiently certain efficacy is available. For this reason, a number of broad-spectrum antibiotics and prophylaxis are often recommended.[4]

Several factors are important in selecting an antibiotic for preoperative prophylaxis. The first consideration is the activity of the agent against poten-

**Address correspondence/reprint requests to Dr. William F. O'Brien, Division of Obstetrics, University of South Florida College of Medicine, Suite 506, 4 Columbia Drive, Tampa, FL 33606.**

Clinical Study

Received January 25, 1994
Accepted May 6, 1994

tial pathogens. Surgical technique and duration of procedure are contributing factors to the overall efficacy. Another determinant is the tissue level attained at the time of bacterial contamination.[5]

As an adjunct to safety and efficacy trials of the combination of piperacillin and tazobactam, we undertook a study to determine the penetration of these agents into gynecologic organs in women scheduled for hysterectomy.

### SUBJECTS AND METHODS

Women in good general health who were scheduled for elective hysterectomy were invited to participate in the study. All of the women were 18 years of age or older, weighed between 47.7 and 95.4 kg, and gave full written informed consent approved by the investigational review boards of the University of South Florida and Tampa General Hospital.

Of 36 potential subjects screened, 24 (67%) were excluded or declined for the following reasons: cancellation of surgery (7); failure to meet inclusion criteria (7); alteration of surgical approach (5); and refusal of subject to volunteer (5). Twelve patients were enrolled but 2 were excluded from analysis: one patient inadvertently received an additional preoperative antibiotic and another was excluded because her samples had been stored at −20°C for a prolonged period of time.

Preoperative screening included medical history, physical examination, chest X-ray, EKG, and laboratory studies (CBC, BUN, total bilirubin, alkaline phosphatase, SGPT, SGOT, creatinine, glucose, uric acid, albumin, and urinalysis). Serum samples were obtained and frozen at −70°C should the need for hepatitis screening arise.

Indications for surgery included leiomyomata (6 cases), endometriosis (2 cases), cervical dysplasia (1 case), and ovarian fibroma (1 case). Salpingo-oophorectomy was performed in 8 of 10 cases and these tissue samples were submitted for analysis.

Subjects ranged in height from 140 to 177 cm, with a mean of 160.5 cm. Weights ranged between 47.7 and 95.4 kg, with a mean of 69.42 kg. Body surface area ranged from 1.41 to 2.0 $m^2$, the mean being 1.72 $m^2$.

A single administration of 3 g of piperacillin and 0.375 g of tazobactam was delivered via infusion pump over 30 min. The individual range for piperacillin was 31.43–62.85 mg/kg and for tazobactam was 3.93–7.86 mg/kg. The mean dose administered was 44.6 mg/kg of piperacillin in combination with 5.57 mg/kg of tazobactam.

Antibiotic infusion began with induction of anesthesia; the average time from the initiation of infusion to the time when circulation to the tissue was interrupted was 1 h. Blood samples were obtained prior to drug administration and at the end of the 30 min infusion. As the uterus, ovary, and fallopian tubes were removed, the time was noted and a blood sample was obtained within 5 min. These were placed on ice until centrifuged, separated into 2 equal aliquots, and stored at −70°C. Similarly, the tissue samples were trimmed of any connective tissue, blotted clean, and weighed before storage. Uterine tissue was separated into endometrial and myometrial components. All serum and tissue samples were stored at −70°C until shipment in dry ice to the sponsor's site for analysis.

Plasma and tissue concentrations were determined by modifications of established methods.[6,7] Separate high-performance liquid chromatographic (HPLC) systems were used for the quantification of each drug. Chromatographic separation of tissue and plasma components was accomplished by reverse-phase separation columns, using column-switching for quantification of tazobactam and isocratic mobile-phase delivery systems for piperacillin. Conventional HPLC has never been sensitive enough to measure β-lactamase inhibitors at concentrations less than 0.5 μg/ml. Analysis of the plasma samples included deproteinization with acetonitrile and cefpodoxime as an internal standard. After removal of the acetonitrile with dichloromethane and centrifugation, the aqueous supernatants were analyzed by HPLC using a column-switching technique that allows a very sensitive and precise measurement of tazobactam in plasma.

The tazobactam concentrations of 10 endometrial, 9 ovarian, and 8 fallopian tube samples were measured. Tissue samples were thawed in ice water. Adhesive blood was blotted away and any tissue parts not identified as endometrium, myometrium, ovary, or fallopian tube were removed. The sample was then transferred to a 3.5 ml polystyrene test tube and weighed on an analytical balance. The weight was noted and the tissue was homogenized in 2 volumes for not more than 10 sec by an ultraturrax homogenizer at 24 × $10^3$ rpm. The resulting homogenate was centrifuged for 10 min at 10 × $10^3$ rpm, and 250 μl of supernatant was

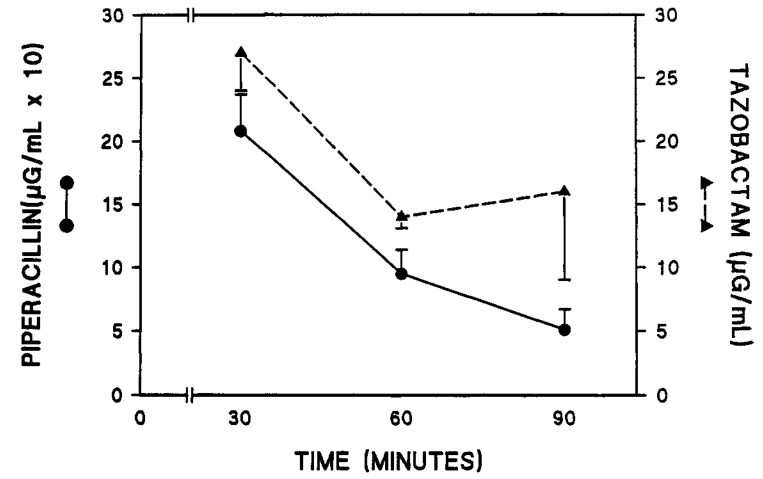

Fig. 1. Plasma concentrations 30–90 min following infusion of 3 g of piperacillin and 0.375 g of tazobactam (mean ± SEM).

deproteinized by the addition of 0.5 ml of acetonitrile containing the internal standard. The acetonitrile was removed by extraction with 1 ml of dichloromethane. After centrifugation at 10 × $10^3$ rpm for 10 min, 120 µl of the aqueous phase was injected onto the HPLC system.

Chromatographic conditions for the mobile-phase analysis were: for precolumn A, 0.1 M of sodium dihydrogen phosphate and 5 mM of tetrabutylammonium hydrogen sulfate; for precolumn B, acetonitrile, A/B: 95/5 (v:v), pH 6.5. The LiChrosorb RP-2, 10 µm (40 × 4.6 mm I.D.), was utilized. The flow rate was 1.0 ml/min and the switching time was 6 min.

For analytical column A, 0.1 M of sodium dihydrogen phosphate and 5 mM of tetrabutylammonium hydrogen sulfate were used. For analytical column B, acetonitrile, A/B: 9/1 (v:v), pH 6.5, was used. The Spherisorb ODS II, 5 µm (250 × 4.6 mm I.D.), was utilized. The flow rate was 1.5 ml/min.

For tazobactam, the retention time was 20–21 min and ultraviolet (UV) absorption was 210 nm. For cefpodoxime, the retention time was 28–30 min and UV absorption was 300 nm. A water bath temperature of 25°C was maintained. The injection volume was 120 µl.

Analysis of the tissue concentrations of piperacillin includes centrifugation and deproteinization with mezlocillin as the internal standard. Conditions for piperacillin measurement were, with the

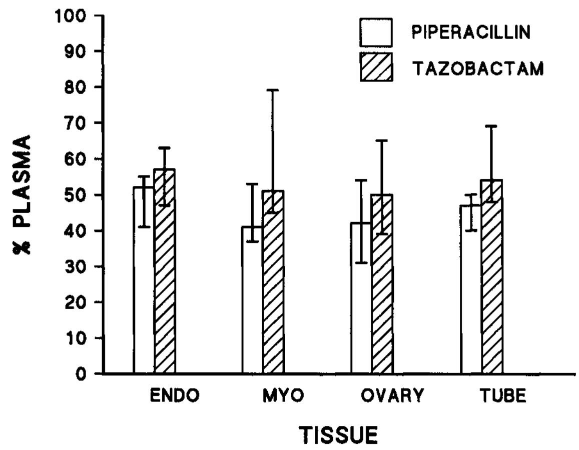

Fig. 2. Tissue concentrations 30–60 min after infusion of 3 g of piperacillin and 0.375 g of tazobactam (median, 25–75 percentile).

mobile phase, column A, 0.1 M of potassium dihydrogen phosphate, and column B, acetonitrile, A/B: 4/1 (v:v), pH 5.5. The LiChrospher C18, 5 µm (250 × 4.6 mm I.D.), was utilized for the analytical column. The flow rate was 1.7 ml/min, the injection volume was 40 µl, and the temperature of the water bath was 37.5°C. UV absorption was 220 nm for piperacillin and for mezlocillin.

Plasma samples were thawed, vigorously mixed, and centrifuged for 5 min at 10 × $10^3$ rpm. The supernatant, 0.100 ml, was stabilized with 0.100 ml of 0.1 M potassium dihydrogen phosphate buffer (pH 5.5) containing the internal standard.

**TABLE 1. In vitro activity of piperacillin/tazobactam (8:1) vs. recent clinical isolates**

| Isolate | No. | MIC 50 | MIC 90 | Range |
|---|---|---|---|---|
| *Escherichia coli* | 2309 | 2.5 | 10.6 | ≤0.5–256 |
| *Enterobacter aerogenes* | 175 | 5.2 | 29.3 | <0.5–128 |
| *E. cloacae* | 481 | 5.3 | 61.0 | ≤0.5–>128 |
| *Klebsiella pneumoniae* | 650 | 1.6 | 17.3 | ≤0.5–128 |
| *K. oxytoca* | 223 | 3.7 | 15.4 | 1–128 |
| *Citrobacter diversus* | 112 | 2.1 | 7.5 | 1–16 |
| *C. freundii* | 121 | 2.0 | 45.1 | 1–64 |
| *Serratia marcescens* | 338 | 6.4 | 25.2 | 0.25–512 |
| *Proteus mirabilis* | 545 | 0.6 | 1.1 | 0.12–64 |
| *P. vulgaris* | 70 | 0.7 | 1.3 | 0.25–64 |
| *Morganella morganii* | 144 | 1.1 | 9.9 | ≤0.5–64 |
| *Providencia rettgeri* | 10 | 128 | 256 | ≤0.5–>128 |
| *P. stuartii* | 31 | 2.5 | 10 | 0.25–32 |
| *Salmonella* spp. | 87 | 7.9 | 49.2 | 0.12–16 |
| *Shigella* spp. | 81 | 3.5 | 47.4 | 0.12–16 |
| *Pseudomonas aeruginosa* | 320 | 5.3 | 57.4 | 0.1–>256 |
| *P. cepacia* | 21 | 10.4 | 14.5 | ≤0.5–128 |
| *Xanthomonas maltophilia* | 135 | 108.6 | 224 | 16–>128 |
| *Acinetobacter* spp. | 354 | 19 | 54.9 | 1–>256 |
| *Aeromonas* spp. | 10 | 2 | 4 | 2–8 |
| *Plesiomonas shigelloides* | 29 | 0.25 | 1 | 0.02–0.06 |
| *Branhamella catarrhalis* | 27 | 0.1 | 0.2 | 0.03–0.12 |
| *Neisseria gonorrhoeae* | 16 | 0.3 | 1.0 | 0.06–2 |
| *N. meningitidis* | 10 | 0.3 | 0.3 | 0.3–0.5 |
| *Hemophilus influenzae* | 53 | 0.2 | 0.4 | 0.06–8 |
| *Staphylococcus aureus*, oxa sensitive | 168 | 3 | 13.6 | ≤0.5–128 |
| *S. aureus*, oxa not specified | 931 | 4 | 8.1 | 1–4 |
| *Staphylococcus* coagulase negative, oxa sensitive | 222 | 1.9 | 3.9 | ≤0.5–32 |
| *Staphylococcus* coagulase negative, oxa not specified | 814 | 2.9 | 22.9 | ≤0.5–128 |
| *Streptococcus pneumoniae* | 93 | 0.3 | 0.5 | 0.03–1 |
| *Enterococcus* | 525 | 3.8 | 8.5 | 50.5–6.4 |
| *Bacteroides fragilis* | 60 | 4 | 16 | <1–32 |

This solution was deproteinized by adding 0.400 ml of acetonitrile. After mixing and centrifugation at $15 \times 10^3$ rpm, the acetonitrile was removed by extraction with 1 ml of dichloromethane. After centrifugation at $10 \times 10^3$ rpm for 10 min, 0.040 ml of the aqueous phase was injected onto the HPLC system.

### RESULTS

Plasma samples were taken from 48 to 96 min post-infusion, corresponding to the time tissues were removed from circulation (Fig. 1). With one exception, the 8:1 concentration ratio remained constant in plasma for up to 96 min after administration. At 95 min, subject 7 had a BSA of 2.0 $M^2$ and a plasma concentration ratio of 5.4.

At the time the tissues were removed from circulation, approximately 1 h after drug infusion, the penetration of piperacillin into gynecological tissues was 40–50% (Fig. 1). The range, 12.8–82.1 mcg/g, was sufficient to ensure antimicrobial β-lactamase activity. Tazobactam penetration was 60–70%, ranging from 2.66 to 12.4 mcg/g. Anti-β-lactamase activity can be assured at these levels.

The range of concentration ratios was broader for tissue samples than for plasma samples. Endometrial tissue had ratios ranging from 4.8 to 8.0, myometrial samples from 3.2 to 9.3, fallopian tube samples from 3.8 to 6.9, and ovarian tissue samples from 4.4 to 8.2 (Fig. 2).

### DISCUSSION

Piperacillin/tazobactam is a recent addition to the growing class of agents that combine broad-spectrum penicillin or cephalosporins with agents that inhibit the activity of β-lactamases. Clinical trials have demonstrated a highly favorable response in several types of infection. Most notably, piperacillin/tazobactam appears to be equally safe and of superior efficacy when compared with imipinem/cilastatin in the treatment of severe intra-abdominal infections.[2] Optimal treatment of gynecologic infections requires broad-spectrum antimicrobial activity in several tissues within the reproductive tract. Tazobactam exhibits the highest affinity for β-lactamases of the currently available inhibitors.[8] When compared with amoxicillin-clavulonate and ampicillin-sulbactam, piperacillin-tazobactam shows the highest activity against β-lactamase containing enteric gram-negative aerobes.[9]

This antibacterial activity, however, must be distributed throughout the tissues of the female genital tract in order to effectively eradicate infection.

In this study, following a single infusion, piperacillin/tazobactam retained an 8:1 ratio in plasma samples obtained from 48 to 96 min following infusion. This stability of proportions was maintained at a moderately consistent level in the tissues studied with a very favorable plasma to tissue ratio of approximately 2:1. In vitro, these concentrations result in effective inhibition against the organisms expected in female genital tract infection (Table 1). This high degree of penetration throughout the tissues of the female genital tract, along with its excellent penetration into areas of inflammation,[1] should result in a high degree of clinical efficacy in the treatment of pelvic infections.

### ACKNOWLEDGMENTS

This study was supported by a grant from American Cyanamid, Pearl River, NY, and the analytical work was carried out by the Institute for Biomedical and Pharmaceutical Research, Heroldsberg/Nurnberg, Germany.

### REFERENCES

1. Wise R, Logan M, Cooper M, Andrews JM: Pharmacokinetics and tissue penetration of tazobactam administered alone and with piperacillin. Antimicrob Agents Chemother 35:1081–1084, 1991.
2. Eklund AE, Nord CE: A randomized multicenter trial of piperacillin/tazobactam versus imipinem/cilastatin in treatment of severe intra-abdominal infections. J Antimicrob Chemother 31:79–85, 1993.
3. Martens MG, Faro S, Maccato M: Susceptibility of female pelvic pathogens to oral antibiotic agents in patients who develop postpartum endometritis. Am J Obstet Gynecol 164:1383–1386, 1991.
4. Peterson B, Walker CK, Kahn JG, et al.: Pelvic inflammatory disease—Treatment issues and options. JAMA 266: 2605–2611, 1991.
5. Stein GE: Patient costs for prophylaxis and treatment of obstetric and gynecologic surgical infections. Am J Obstet Gynecol 164:1377–1379, 1991.
6. Marunaka T, Maniwa M, Matsushima E, Minami Y: High-performance liquid chromatographic determination of a new β-lactamase inhibitor and its metabolite in combination chemotherapy with piperacillin in biologic materials. J Chromatogr 431:87–101, 1988.
7. Ocampo AP, Hoyt KD, Wadgaonkar N, et al.: Determination of tazobactam and piperacillin in human plasma, serum bile and urine by gradient elution reversed-phase high-performance liquid chromatography. J Chromatogr 469:167–179, 1989.
8. Culmann W: Beta-lactamase inhibitors: Relation between kinetic data and in-vitro synergism studies. Int J Med Microbiol 275:500–503, 1991.
9. Gatermann S, Marre R: Comparative in vitro activities of amoxicillin-clavulonate, ampicillin-sulbactam, and piperacillin-tazobactam against strains of *Escherichia coli* and *Proteus mirabilus* harbouring known beta-lactamases. Infection 19:106–109, 1991.
